# Promoting More Equitable Global Health Research, Education, and Community Partnerships: The Efforts of One US‑Based Academic Institution

**DOI:** 10.5334/aogh.4772

**Published:** 2025-06-11

**Authors:** Sarah Emoto, Laura Ferguson, Lourdes Baezconde-Garbanati, Howard Hu, Goleen Samari, Sofia Gruskin

**Affiliations:** 1 Institute on Inequalities in Global Health, Keck School of Medicine, University of Southern California, Los Angeles, CA, USA; 2 Department of Population and Public Health Sciences, Institute on Inequalities in Global Health, Keck School of Medicine, University of Southern California, Los Angeles, CA, USA; 3 Department of Population and Public Health Sciences, Keck School of Medicine, University of Southern California, Los Angeles, CA, USA

**Keywords:** global health, global health partnerships, equity

## Abstract

*Objectives:* Equitable global health partnerships are recognized as critical for health equity; however, power imbalances and structural inequities continue to undermine these partnerships and ultimately their ability to achieve equitable health outcomes. As individuals within a US‑based academic institution engaged in global health partnerships during the current, complicated political moment, we recognize our responsibility to critically examine what it means for us to seek to engage equitably, both locally and globally. We therefore undertook an initiative to develop and adopt a set of principles to serve as internal guidance for how individuals within our institution engage in partnerships. We present our approach to promoting equity within our local and global research, education, and community partnerships, informed by existing literature, as an example of how academic institutions based in the Global North might seek to address power imbalances and to engage with others involved in similar efforts.

*Methods:* We reviewed similar initiatives and existing principles. An internal, departmental committee coalesced around eight principles and drafted and iteratively refined their components. As part of ongoing, broader conversations with our partners, during this process, we engaged and sought the perspectives of our external partners, including from Brazil, India, and Kenya, to ensure the guidance was both informed by and resonated with their concerns about engaging with a US‑based institution.

*Findings:* The principles of sustainability, mutual benefit and reciprocity, equitable governance, do no harm, locally identified priorities, compliance with ethical reviews and legal standards, information sharing, and accountability were elaborated and ultimately adopted by the full department faculty.

*Conclusions:* Despite the best of intentions, we foresee challenges that may impact both implementation and outcomes. Continued reflection and dialogue with our partners and others engaged in similar initiatives is needed to address these challenges and the broader structural inequities embedded in global health.

## Introduction

### Background

Equitable global health partnerships are understood as essential for achieving health equity. Experience has also demonstrated they are critical for advancing the strength and sustainability of population health improvements [1]. However, in practice, power imbalances continue to undermine partnerships and the effectiveness of their work. There is increasing attention to the issues contributing to power imbalances and the need to address them [2–10]. These issues are situated within a broader discourse on decolonizing the field of global health, given its colonial legacy and persisting structural inequities [11–15]. Within global health partnerships, imbalances often occur between partners based in the Global North and those based in the Global South. Acknowledging this geography‑based terminology is imprecise, we use it as shorthand to signal power imbalances, recognizing imbalances also occur across other intersecting dimensions of position and power of individuals and institutions, between and within countries [13, 16]. There is also increasing momentum to respond and change how partnerships operate. As individuals based at a US academic institution who engage in global health research, education, and community partnerships, these issues have never been more salient for us, particularly in the current political moment with the changing US global health funding landscape since January 20. We recognize the responsibility and complexity of critically examining what it means for us to seek to engage equitably in all partnerships, locally and globally. We undertook an initiative to develop and adopt a set of principles to serve as internal guidance for how individuals within our institution engage with partners in collaborative work, informed by existing literature.

### Aim

The overall aim of this paper is to share our approach to promoting more equitable research, education, and community partnerships as one example of how an academic institution in the Global North may seek to address power imbalances. Acknowledging that such efforts may vary based on institutional contexts and internal politics and processes, we aim to engage in dialogue with other institutions and individuals around the world involved in similar efforts, in the hopes of generating useful discussion and change. This paper does not suggest that adoption of the principles presented constitutes a single solution to the systemic inequities that plague partnerships and global health more broadly. Instead, we see this as an opportunity to build upon existing work and develop a set of principles, drawing on broadly accepted concepts and tailored to our institutional context.

## Development of Principles

### Objective of the initiative

This initiative emerged based on ongoing conversations with long‑standing partners in many parts of the world. Recognizing there are different approaches to the development and scope of such principles, including joint efforts and application across multiple institutions, through our conversations, it became apparent that we additionally were in need of an internal process and guidance, as a means of conducting our work and holding ourselves and our institution accountable. The work under this initiative began as a collaboration between the Institute on Inequalities in Global Health (IIGH) and the Department of Population and Public Health Sciences (PPHS), both housed within the Keck School of Medicine at the University of Southern California (USC). This collaboration was based on a shared concern and joint vision for promoting equity in global health partnerships, from within our US‑based institution. The initial objective was to adopt principles and actionable guidance applicable to our institution’s research, education, and community partnerships with any type of organization, academic, government, non‑governmental, or otherwise. As global health encompasses our work in all geographies and various systems, we sought to capture principles applicable to both local and international partnerships.

The intended users for the principles are IIGH and PPHS faculty, staff, students, and volunteers. The principles are additionally intended to clarify how we intend to work for the partners with whom we engage.

### Review of existing principles and guidelines

A range of global health organizations have undertaken efforts to address inequitable partnerships, and several sets of principles and guidelines exist. We identified existing principles, guidelines, and descriptive literature from iterative searches of peer‑reviewed articles and gray literature, as well as manual searches of other universities’ websites to identify applicable principles and concepts. We included examples for review if they contained content relevant to global or local health research, education, or community partnerships.

Some principles identified appeared to be specific to different types of international partnerships, such as short‑term educational experiences abroad [17–19] or certain types of research collaborations [16, 20, 21]. Some guidelines explicitly focus on north–south partnerships [22–25]. Target audiences also varied, for example, with some directed toward researchers within a specific field in global health, such as epidemiology [21], while others offer guidance for academic health science departments broadly [26]. A scoping review of guidelines undertaken by Voller and colleagues identified that some do not specify the target audience or state generally that they are intended for “researchers”, which the authors note may impact implementation, as it may be unclear to whom they apply [22]. Some academic institutions also have principles and guidelines related to their global engagement publicly available on their websites [27–30]. Given our intent for principles to encompass both our local and global partnerships, we also looked to principles of community engagement [31–34]. We also noted existing tools for evaluating partnerships, which may be useful as we operationalize the principles [7, 35, 36].

We also reviewed existing principles for the process and approach used to develop them. Most initiatives from other Global North academic institutions, as described on their websites, involved convening faculty committees. In the literature, while most examples originate from institutions in the Global North, the processes frequently referenced consultation with partners in the Global South. We identified few examples originating from the Global South [26, 37, 38]. We found the approach taken by a committee convened in Uganda, which included sending the committee’s draft recommendations to an external review panel for feedback, to be of particular relevance to our process [37].

Our review did not uncover a single set of principles that comprehensively addresses research, education, and community partnerships, applicable to both local and global contexts, and providing enough specificity for practical implementation. Therefore, we drew on various examples, both in process and content, to adapt existing concepts into a set of principles aligned with our objectives and tailored for application within our institution.

### The process of drafting the principles

After compiling a review of existing principles, a drafting committee was formed of faculty experienced in global and local community health. Informed by the literature and others’ initiatives, the committee identified eight principles they agreed captured the categories of concepts determined to be applicable to promoting equitable local and global research, education, and community partnerships. Over the course of a year, the committee engaged in an iterative process of identifying, discussing, drafting, and refining iterations of these principles, including detailed components and actionable guidance for each. During this process, the committee engaged and sought feedback on the draft document from external partners, including from Brazil, India and Kenya. This external consultation was one aspect of broader and ongoing conversations with partners about the need for such guidance, and was a critical step to ensure that the principles and guidance were both informed by and resonated with external perspectives. Given the aim of this paper to share the internal, institutional approach to adopting principles within IIGH and PPHS, our external partners are not co‑authors on this paper focused on our internal processes. Once the document sat comfortably with the committee and our external partners, review and approval by additional department leadership were sought, and the final document was signed off and adopted by the full 127 department faculty across six divisions.

## Principles of Local and Global Engagement

In this section, we introduce overarching elements of the principles and then describe the eight principles we identified and determined to be most relevant for our purposes, alongside some of the issues raised in the literature to illustrate the types of imbalances each aims to address. We then elaborate on the components we understand the application of each principle to encompass in practice. Table 1 presents the adopted principles with key components. The full document of the principles, which includes additional details, recommendations, and examples of actions corresponding to various key components, is available online [39].

**Table 1:** USC, IIGH, and PPHS principles of local and global engagement with key components.

PRINCIPLE	KEY COMPONENTS
Sustainability	Commitment to long‑term relationships Shared vision and long‑term goals Understanding of existing partnerships Commitment to supporting other collaborations
Mutual benefit and reciprocity	Mutual benefit Reciprocal opportunities Mutual and inclusive participation Bidirectional communication Mutual respect Reciprocal learning and capacity building Mutual engagement with budgets Equitable authorship
Equitable governance	Recognize and address power dynamics Shared and inclusive decision‑making Bidirectional and inclusive communication strategies Transparent decision‑making Clear structures for governance Transparent and equitable resource management Transparent and ongoing review of the partnership governance structures
Do no harm	Prevention of unintended consequences Awareness of long‑term impacts Awareness of political, legal, and social environments
Locally identified priorities	Engagement of local partners Alignment with local priorities Awareness of current policy landscape Awareness of existing work
Compliance with ethical reviews and legal standards	Ethical conduct Approval from all appropriate ethics boards Respect for community rights Awareness and understanding of laws and implications
Information sharing	Equitable and transparent data ownership Equitable dissemination to communities Accessible dissemination Institutional awareness within the university
Accountability	Accountability for evaluation of programs Accountability to communities and individuals impacted Accountability to other partners Accountability to the partnership

### Overarching elements

The committee discussed and agreed upon several overarching elements of all principles. These include the principles’ interdependence, alignment with international human rights norms, and the commitment to continually work toward justice, equity, diversity, inclusion, and accessibility through all aspects of partnerships. Each principle is briefly described below.

### Sustainability

Within the literature reviewed, sustainability consistently emerged as a key concept, in particular the need to shift from short‑term objectives and engagement toward long‑term relationships with sustainable impacts [4, 7, 16, 26]. Partnerships that lack long‑term investment or institutional commitments are likely to have limited impact on strengthening the capacities of all partners, and thus limited impact on health systems, communities, or health outcomes over the long term [3, 7]. We identified commitment to long‑term relationships, a shared vision with long‑term goals, an understanding of existing partnerships, and support for a range of collaborations among partners and networks as key to the practice of operationalizing sustainability.

### Mutual benefit and reciprocity

Mutual benefit and reciprocity appear within the literature as critical to ensuring that partnerships neither disproportionately benefit nor burden any one partner and operate reciprocally [16, 25, 26, 38]. Unevenly distributed benefits are often recognized as contributing to and resulting from power imbalances. Identified examples of benefits that may be unequally shared include, but are not limited to, access to and control of funding or other resources, prioritization of one partner’s interests over another’s, and authorship [3–5]. In order to support more balanced partnerships in practice, we consider the application of mutual benefit and reciprocity critical through co‑defined actions that underscore mutual respect and mutual agreement on reciprocal benefits, including in relation to opportunities, participation, capacity building, budgets, and authorship.

### Equitable governance

Equitable governance structures, including shared and participatory decision‑making, are often cited as key to addressing inequitable partnerships [3, 23, 40]. Power imbalances within governance can stem from myriad factors, including a lack of transparent communication, imbalance in budgets, including how resources are allocated and who is hired, undefined roles among partners, and inequalities in agenda‑setting and decision‑making [4, 5]. The literature and committee discussions identified the importance of recognizing and addressing both the realities and appearances of unequal power dynamics, rather than maintaining a pretense of equality when asymmetries are apparent or present [40, 41]. This requires consistent and sustained effort by all partners. Openly engaging in conversations to address power dynamics over time enables addressing other key governance aspects, including shared and transparent decision‑making and resource management, bidirectional communication, clear and transparent structures, and ongoing review of partnership governance structures.

### Do no harm

The responsibility to do no harm is widely accepted in health research. In the context of global health partnerships, this approach is often understood to extend beyond mitigating clinically related risks to ensuring that the implications of broader contexts and potential risks to all individuals, communities, and partners involved are carefully considered throughout each project stage [20, 27, 28]. While those engaged in global health ostensibly do not intend to cause harm, “unintended harms” that occur often relate to social, political, or legal factors that were not sufficiently considered in project design or implementation and may have resulted in increased risks, particularly for vulnerable communities [42, 43]. In applying this principle, we understand it to include attention to preventing unintended consequences and addressing harms that do occur, consideration of long‑term impacts, and mutual understanding of the political, legal, and social environments within which the partnership and its programs take place.

### Locally identified priorities

Within the literature, ensuring the work that partners undertake aligns with and addresses locally identified priorities is consistently identified as important for equitable partnerships [17, 19, 22, 40]. When programs misalign with local priorities, they may be unsustainable, duplicate, conflict or interfere with existing work, or redirect critical resources away from real priorities. We interpret this principle to encompass the active engagement of researchers and local partners in defining a project’s scope, which includes identifying, aligning with, and addressing local interests, needs, and concerns, as well as adjusting priorities as necessary based on community feedback. Additionally, staying aware of current and evolving national and local policies is essential to contextualize agreed‑upon work plans that both reflect locally identified priorities and consider broader strategic objectives.

### Compliance with ethical reviews and legal standards

While ethical standards are inherently relevant to all principles for promoting equitable engagement, including, as they relate in the first instance, to “do no harm,” compliance with ethical review and legal standards additionally appears in the literature as a broader and distinct concept. Often cited examples highlight the need to address the ethical and legal issues raised when, for example, research is conducted without approval from local ethics boards, or when medical students and volunteers from Global North institutions practice clinical care in Global South settings, which they could not do at home without appropriate training or licensure [17, 26, 38, 44]. To address these issues and in alignment with “do no harm,” we understand this principle to encompass all parties maintaining the highest standards of ethical conduct, seeking approval from all appropriate ethics boards and local authorities, respect for community rights, and consistent monitoring of changing legal environments, alongside review and understanding of relevant laws and regulations, with attention to those that may be harmful, and the implications and potential risks for all participants or partners.

### Information sharing

The literature broadly identifies information sharing as crucial for fostering equitable partnerships, emphasizing that information should be available, accessible, and appropriately disseminated within the partnership, while also ensuring that findings are shared with the individuals and communities involved and impacted [21, 22, 40]. Inequitable data access and ownership, including practices such as removing biological samples from countries in which the data were collected, have long been understood to contribute to partnership imbalances [4, 5, 38, 40]. Further imbalances are well known to occur when communities that were engaged in research, whether as participants or observers, or who are impacted by the results, are not informed of the findings [7, 20, 22]. Thus, we consider information sharing to include equitable and transparent data ownership with our partners, alongside equitable dissemination to affected communities. Based on committee discussions, we also understand that information sharingmeans raising institutional awareness, including sharing information with wider university and community partners, alongside others engaged in research, education, or community programs in the same community or geography.

### Accountability

Accountability is often presented in the literature as a distinct principle and one that underpins how other principles operate in practice, encompassing shared accountability within the partnership as well as accountability to the communities impacted, to funders, and to other relevant actors [5, 17, 20, 26]. Lack of accountability and/or accountability structures that only operate in one direction, or simply with attention to funder requirements, may result in imbalances and mistrust among partners, or between partners and other actors [5]. We thus consider accountability as an ongoing and iterative process that includes the monitoring of how programs are conducted, evaluation of the program’s impact on communities and individuals, accountability to other relevant actors, and evaluation of all aspects of the partnership itself.

## Challenges

While the adoption of these principles is a positive step, we foresee implementation challenges in practice, including within partnerships, within institutions, and in how the principles interact with broader global health structures, including the geopolitical context within which partnerships operate. Our intent was to adopt principles similarly applicable to the research, education, and community partnerships with which our institution engages; however, it will be useful going forward to understand what, if any, nuances may impact their generalizability across these domains. We understand that the application of these principles in practice requires an awareness of the types of challenges noted below in order to address them and work toward the intended outcome of addressing structural power imbalances.

### Challenges within partnerships

First, while these principles were adopted as internal guidance for individuals within our own institution, we recognize, even with consultation with external partners, the inherent limitation in developing these principles as individuals at an academic institution based in the Global North, and we will seek to ensure ongoing reflection internally and with our partners on the application of these principles in the context of our respective positions and identities. As part of continuing dialogue with others involved in similar work, considering how these principles align with other institutions’ efforts to promote equitable partnerships, including those based in the Global South [37] and non‑academic institutions, is one way we hope to practice this reflection.

A critical point made by external partners during their review and feedback was the importance of operationalization, the actions taken to go beyond a commitment on paper that would lead towards real shifts in practice. Thus, we anticipate challenges down the road as, in working with our partners, we identify issues that arise in operationalizing and evaluating the principles. Additional steps, including developing and utilizing existing tools to evaluate partnerships, are likely needed [7, 35, 36].

### Institutional challenges

The principles, as adopted by the full department faculty, exist as voluntary guidance, even as that may limit sustained engagement and accountability even within the department. While enforcement and concrete accountability policies also warrant consideration, the voluntary nature of adoption may be a necessary step in gaining the necessary traction within an institution, particularly based in the Global North, to take on such an initiative, which may in turn pave the way for future policy changes.

The principles were developed with a localized approach focused on department level application. To extend their application beyond a single department, expanding the scope to include the broader school of medicine, and potentially the entire university, would be ideal but may require additional reflection. Challenges may also arise in securing support for adopting these principles at the school and university levels. This initiative initially encountered resistance from some faculty at the department level, and consequently, the voluntary nature was important for buy‑in. Navigating politics at higher levels is likely to be even more complex and require engagement with multiple internal policy structures and entities.

Other institutional factors may also impact how such principles are applied in practice. For example, metrics for promotion at academic institutions that require lead authorship are known to incentivize individuals to prioritize their own position, which may compromise mutual benefit and equitable authorship [45]; formal recognition does not yet exist for authors who build capacity and support others to become lead authors. This may have differential impacts depending on both individual and institutional positionalities, roles, and power dynamics, whether between or within countries. It is important that academic institutions consider the range of policies and practices that may interfere with the full adoption of such principles, with efforts to harmonize approaches and obtain larger institutional buy‑in —all with an eye towards supporting the intended functioning and outcomes of equitable partnerships.

### Challenges in broader global health structures

The constraints imposed by broader global health structures must also be examined, with attention to how to address those that may undermine application in practice. For example, funding mechanisms continue to limit indirect costs to partners, fund only short‑term projects, or require a principal investigator to be based in a high‑income country [13, 22, 46]. Such structures conflict with principles that aim to promote long‑term and sustainable partnerships, mutual benefit, and equitable governance, but remain part of the day‑to‑day realities of the global health industry. With the architecture of global health increasingly constrained by limited funding, it is important to consider opportunities to address these realities and shift broader norms, encompassing requirements imposed by actors beyond academic institutions, such as governments, private donors, and academic publishers [13]. As individuals at a US‑based institution, we recognize the gravity and complexity of the current political moment, and consider the adoption of these principles and addressing these broader challenges to be all the more important.

## Conclusion

The principles described in this paper are newly adopted and represent just one example of an approach taken by an academic institution in the Global North, with a view to adopting internal guidance as a means of holding ourselves accountable to engaging more equitably in local and global health partnerships. A broader dissemination plan within our university is in progress, with an operational plan to follow. This may include initiatives such as training workshops to support faculty, staff, and students in applying these principles. Recognizing the evolving nature of global health, the principles are intended to serve as a living document, open to review, evaluation, and revision. While the adoption of these principles alone cannot resolve widespread structural inequities, we present them as a contribution to ongoing dialogue and efforts to critically examine and advance equitable partnerships. Building on others’ momentum and aligning with the broader imperative to decolonize global health, we encourage and welcome ongoing engagement, critique, and discourse on various approaches to foster more equitable partnerships. Given the current political environment, including the massive shifts in funding due to geopolitical priorities, it is all the more important that equity is prioritized in all aspects of engagement. Ultimately, this will help individuals and institutions to be better positioned to leverage global health partnerships toward achieving health equity for all.

## Data Availability

Not applicable—no datasets were generated and/or analyzed for this work.
